# Single and combined impacts of irradiation and surgery on lymphatic vasculature and fibrosis associated to secondary lymphedema

**DOI:** 10.3389/fphar.2022.1016138

**Published:** 2022-10-18

**Authors:** F. Buntinx, A. Lebeau, L. Gillot, L. Baudin, R. Ndong Penda, F. Morfoisse, F. Lallemand, G. Vottero, C. Nizet, J. L. Nizet, S. Blacher, A. Noel

**Affiliations:** ^1^ Laboratory of Tumor and Development Biology, GIGA-Cancer, University of Liège (ULiège), Sart-Tilman, Liège, Belgium; ^2^ U1297-Institut des Maladies Métaboliques et Cardiovasculaires (I2MC), Institut National de la Santé et de la Recherche Médicale (INSERM), University of Toulouse, Toulouse, France; ^3^ Department of Radiotherapy-Oncology, Centre Hospitalier Universitaire (CHU) de Liège, University of Liège, Liège, Belgium; ^4^ Department of Plastic and Reconstructive Surgery, Centre Hospitalier Universitaire (CHU) de Liège, University of Liège, Liège, Belgium; ^5^ Walloon Excellence in Life Sciences and Biotechnology (WELBIO), Wavre, Belgium

**Keywords:** lymphedema, irradiation, fibrosis, lymphatic, periostin, tenascin-C, collagen

## Abstract

Lymphedema (LD) refers to a condition of lymphatic dysfunction associated with excessive fluid accumulation, fibroadipose tissue deposition and swelling. In industrialized countries, LD development mainly results from a local disruption of the lymphatic network by an infection or cancer-related surgery (secondary LD). In the absence of efficient therapy, animal models are needed to decipher the cellular and molecular mechanisms underlying LD and test putative drugs. In this study, we optimized and characterized a murine model of LD that combines an irradiation of the mice hind limb and a radical surgery (lymph node resection associated to lymphatic vessel ligation). We investigated the respective roles of irradiation and surgery in LD formation by comparing their impacts, alone or in combination (with different intervention sequences), on eight different features of the pathology: swelling (paw thickness), indocyanine green (ICG) clearance, lymphatic vasculature remodeling, epidermal and dermal thickening, adipocyte accumulation, inflammatory cell infiltration and collagen deposition. This study supports the importance of radiation prior to surgery to experimentally induce a rapid, severe and sustained tissue remodeling harboring the different hallmarks of LD. We provide the first experimental evidence for an excessive deposition of periostin (POSTN) and tenascin-C (TNC) in LD. Through a computerized method of digital image quantification, we established the spatial map of lymphatic expansion, as well as collagen, POSTN and TNC deposition in papillary and reticular dermis of lymphedematous skins. This mouse model is available to study the patho-physiology of LD and test potential therapeutic targets.

## Introduction

The lymphatic system is a blind and hierarchical vascular network composed of absorptive and conducting vessels whose functions include the maintenance of tissue fluid balance, immune cell trafficking and dietary lipid absorption (Swartz, 2001; Aspelund et al., 2016; Betterman and Harvey, 2016; Petrova and Koh, 2018; Oliver et al., 2020). Excessive growth of lymphatic vessels (lymphangiogenesis) is associated with transplant rejection and cancer (Vaahtomeri et al., 2017; Morfoisse and Noel, 2019). Failure of the lymphatic system leads to an impaired drainage capacity resulting in fluid accumulation and tissue swelling called lymphedema (LD) (Grada and Phillips, 2017; Dayan et al., 2018; Azhar et al., 2020; Brown et al., 2022). LD is a chronic, multifactorial disease affecting approximately 250 million people worldwide (Oliver et al., 2020). It is broadly categorized as primary forms (congenital) often resulting from mutations in genes involved in lymphatic development (Brouillard et al., 2021), or secondary forms reflecting structural and functional insults to the lymphatic vasculature due to infection (lymphatic filariasis) or tissue damage (Azhar et al., 2020). Currently, secondary LD is the most common form in developed countries, which occurs after cancer treatment (radiotherapy and/or surgical lymph node dissection) (Kurt et al., 2016). It is estimated that 20–30% of cancer patients develop LD after treatment, mainly in breast, gynecological, melanoma, prostate, testis, head and neck cancers (Garfein et al., 2008; Shigaki et al., 2013; Rockson, 2019a). Although not fatal, LD affects the quality of life due to mobility disruption of the affected limb, pain, psychological morbidity (poor self-image) and increased risk of skin infections (Grada and Phillips, 2017). This lifelong and unpredictable condition often develops in a delayed manner, months or even years after surgery. Current treatments are poorly effective, mainly palliative and include compression massage, extensive decongestive therapy by physiotherapists, restrictive diets or surgery to transfer vascularized lymph nodes (Carl et al., 2017; Borman, 2018; Chang et al., 2021; Brown et al., 2022). Pharmacological treatments based on pro-lymphangiogenic factors (VEGF-C) are promising, but are not sufficient to restore functional lymphatic vessels with efficient valves (Szoke et al., 2021; Brown et al., 2022). Intensive research is thus still needed to design the ideal treatment for LD.

To study this multifactorial disease and decipher its underlying molecular and cellular mechanisms, accurate *in vivo* models are required to reproduce all LD features including fluid accumulation, limb swelling, skin thickening and subcutaneous fibro-adipose tissue deposition (Shin and Rockson, 2008; Rockson, 2019b). Although large animal models have been described, mouse models remain the most widely used models due to their reproducibility, cost and applicability to transgenic mice (Shin et al., 2003; Rockson, 2019b; Hassanein et al., 2021). In order to mimic the development of cancer-associated LD resulting from a cascade of injuries, local irradiation of the lower limb can be combined to node ablation and lymphatic vessel ligation. Impaired lymphatic formation and function has been long assumed as the primary event, sufficient to initiate LD. If this holds true, one would expect LD to develop in all patients. However, recent studies have revealed the crucial contribution of chronic inflammation-induced fibrosis in LD (Kataru et al., 2019). Fibrosis in LD mainly refers to the accumulation of thick collagen bundles surrounding lymphatics. However, the non-collagenous matrix composition of LD-associated fibrotic tissue, as well as the interplay between fibrosis and lymphatic vascular remodeling remain elusive. The presence of two non-structural matricellular proteins, periostin (POSTN) and tenascin-C (TNC), have been detected in human lymphedematous samples through transcriptomic analyses (Planck et al., 2011). No data are yet available for these matricellular proteins in experimental LD setting and at the protein levels. We here optimized and characterized a murine model combining a local irradiation of the inguinal region and surgery (lymph node ablation and lymphatic vessel ligation). We evaluated the impact of surgery and irradiation (administrated alone or in two sequential combinations) on the formation of LD (paw thickness and indocyanine green clearance) and their histological features including lymphatic vasculature, epidermis hyperplasia, dermis thickening, the organizational pattern of collagen fibers (thin and thick fibers), POSTN and TNC deposition. Computer-assisted image analyses were applied for quantification.

## Materials and methods

### Mice

The experiments were performed using adult female (6–8 week old) on Fvb/N mice (Aspelund et al., 2016). The animals were housed in light- and temperature-controlled environments and fed *ad libitum*. All animal experiments were approved by the Institutional Animal Care and Use Committee at University of Liege (20-2204)*.* The total number of mice enrolled in the study was 48 (8–12 per experimental group).

### Hind limb LD induction model

For the local irradiation of the left limb, mice were anesthetized with 2% isoflurane and placed on their ventral side inside the X-Rad SmART PXi Precision X-RAY irradiator (GE Healthcare, Belgium). An X-ray radiography of the mouse (40.0 kV, 0.5 mA) was done to precisely target the inguinal region. A 20 mm square collimator was then placed to irradiate locally the left limb by a single dose of 30 Gy (225.0 kV, 13.00 mA, 2 × 317 s). Mice were irradiated for 317 s on the dorsal thigh side and 317 s on the ventral thigh side for a total administered dose of 30 Gy. Mice were operated under 2% isoflurane gas anesthesia and under a horizontal airflow hood. Before surgery, the lower back of mice and the left paw were shaved and disinfected with dermal isobetadine. Blue Evans (5 µL) was injected between the footpads of the left hind foot paw to visualize lymphatics. Surgery was performed in three steps: 1) a circumferential incision of the skin at the inguinal level, 2) an excision of the inguinal and popliteal lymph nodes and finally, 3) a ligation of collecting lymph vessels parallel to the ischial vein by three separate points of 7/0 non-absorbable polypropylene suture (ETHICON, Scotland) under a binocular microscope (×10 magnification). At the end of the procedure, the skin was sutured with separate stitches of 5/0 non-absorbable silk suture (ETHICON, Scotland). Bilateral paw thickness was measured every 3 days using a caliper. To account for individual variability, paw thickness was normalized for each animal by calculating the percent change relative to the control (unoperated) paw using the formula (T/Tc) x100, where T is the thickness of the operated paw and Tc is the thickness of the control paw. For all animals, only the left limb was subjected to irradiation and/or surgery, while the right limb was used as an internal control. Mice were sacrificed 28 days after the first intervention (surgery or irradiation) in all groups.

### *In vivo* indocyanin green fluorescence imaging

Mice were anesthesized with 2% isoflurane and positioned on their ventral side inside an IVIS Spectrum (Xenogen, Caliper Life Sciences). The imaging parameters were: λex = 745 nm, λem = 840 nm, exposure time 0.1 s, f/stop 2, medium binning, field of view 6.6 × 6.6 cm^2^. Five µL of Indocyanin Green (ICG) dye (Verdye, 0.1 mg/ml) were intradermally injected into each mouse hind foot paw. Immediately after injection, serial images were acquired at different times (time 0, 1, 3, 5, 7 h). Living Image software (Caliper Life Sciences) was used for image analysis. Regions of interest (ROI) were delineated over the hind foot paws. Average signal intensity values were recorded for each ROI and plotted versus time in GraphPad Prism 9.0 software.

### Histological examination and (immuno)-histochemical staining

After sacrifice, the fur was removed from hind paw using topical hair remover cream. Paw skin tissues were collected, fixed in 37% formalin and embedded in paraffin. Sections of 5 µm were cut using microtome and three tissue sections spaced 50 µm in paraffin block are placed on each slide. Slides were deparaffinized/rehydrated. For collagen evaluation, samples were sliced (5 μm thickness) and stained with Sirius red and Masson’s trichrome according to the standard procedure (Gardenier et al., 2017). For Sirius red, all sections were digitalized using an Olympus system (polarized light). From the Masson’s trichrome stained sections, the analysis of the epidermis and dermis thicknesses was performed by five independent measurements taken with ImageJ on at least two sections per mouse (*n* = at least seven mice for the different conditions). For immunohistochemistry, antigen unmasking was performed for 11 min at 126°C in citrate buffer (pH 6). Non-specific antigen blocking was performed using protein block buffer. Primary antibodies were incubated overnight at 4°C. We used the following primary antibodies raised against: POSTN (1:200, AG-20B-0033B, Adipogen), CD45 (1:200, Ab10558, Abcam), TNC (1:200, AB19011, Chemicon) and Lyve-1 (1:200, AF2125, R&D Systems). The following secondary antibodies were used diluted to 1:200 and were incubated 1 h at room temperature: donkey anti-mouse, donkey anti-rabbit, donkey anti-goat conjugated to Alexa Fluor 488 or to Alexa Fluor 555 (Invitrogen). Nuclei were stained and slides were mounted in DAPI fluoromount. All images of section immunostaining were acquired using ×20 magnification with the NanoZoomer 2.0-HT system and using the NDP. view software.

### Computerized image analyses

Digital images of whole tissue sections were acquired at ×20 magnification with the NanoZoomer 2.0-HT system (0.23 μm/pixel, scanning resolution). Computerized quantifications were conducted on a minimum of 21 images per experimental conditions. Image processing and quantification of the various staining’s were performed using the images analysis toolbox of MATLAB R2021b, according the following steps (Blacher et al., 2008; Balsat et al., 2014; Blacher et al., 2016): 1) original images were registered in the full-color red, green, blue (RGB) space; 2) contrast was enhanced by calculating the excess of each color (for example by performing 2*R-G-B, for the red component); 3) the resulting images were binarized using an automatic thresholding technique, and then systematically compared to the original ones and corrected manually if required. The spatial distribution of stained regions in relation to the basal epithelial layer was determined as previously described (Balsat et al., 2014; Balsat et al., 2017). Mean spatial distribution curve was then analyzed according the following parameters: 1) the area under the curve referred to as “integrated area”, which gives the global extent of the stained region, 2) the distance (from the epithelial basal layer) up to which 90% of staining was detected (90th percentile = P_90_). Distribution curves of Control and LD determined for each condition were compared point by point using Mann-Whitney non-parametric test.

### Statistical analysis

Statistical analysis was performed with GraphPad Prism 9.0 software using Mann-Whitney non-parametric test, non-parametric Kruskal-wallis with Dunn’s multiple comparison or two-way ANOVA with multiple comparison (Tukey’s test), as indicated in the figure legends. Data are shown as median ± interquartile range and differences were considered statistically significant when *p* < 0.05, as indicated by asterisks with *p* < 0.05 (*), *p* < 0.01 (**), *p* < 0.001 (***) and *p* < 0.0001 (****).

## Results

**FIGURE 1:**
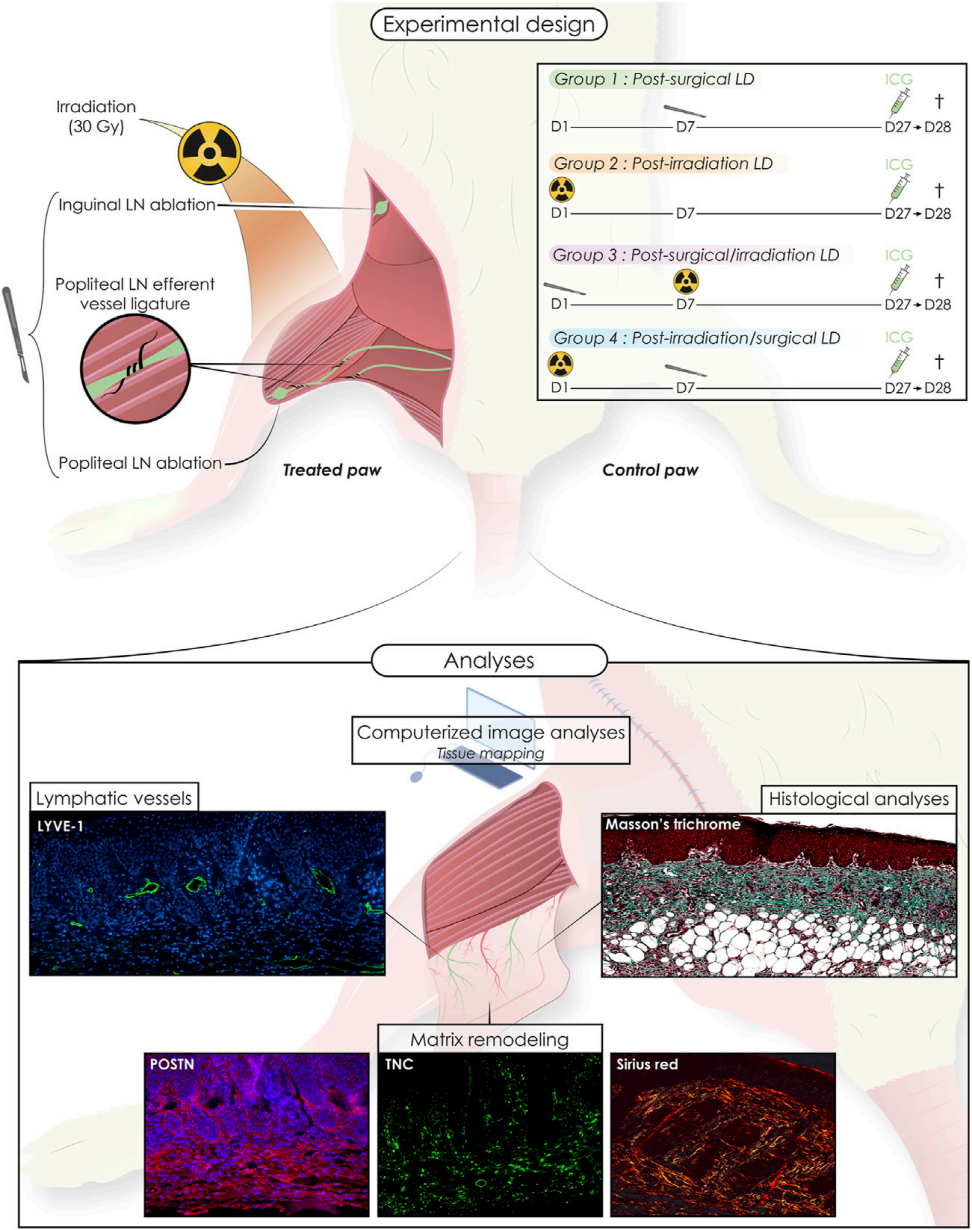
Schematic representation of the mice experimental model of secondary hind limb lymphedema (LD) induced by surgery and/or irradiation (alone or combined, in two sequences of intervention). Animals were separated into four groups: post-Surgery (*n* = 8), post-Irradiation (*n* = 7), post-Surgery/Irradiation (*n* = 11), post Irradiation/Surgery (*n* = 13). For each animal, the left hind limb was subjected to LD induction, while the right hind limb was used as control. The chronology of the interventions is detailed for each group as well as the different analyses performed.

**FIGURE 2:**
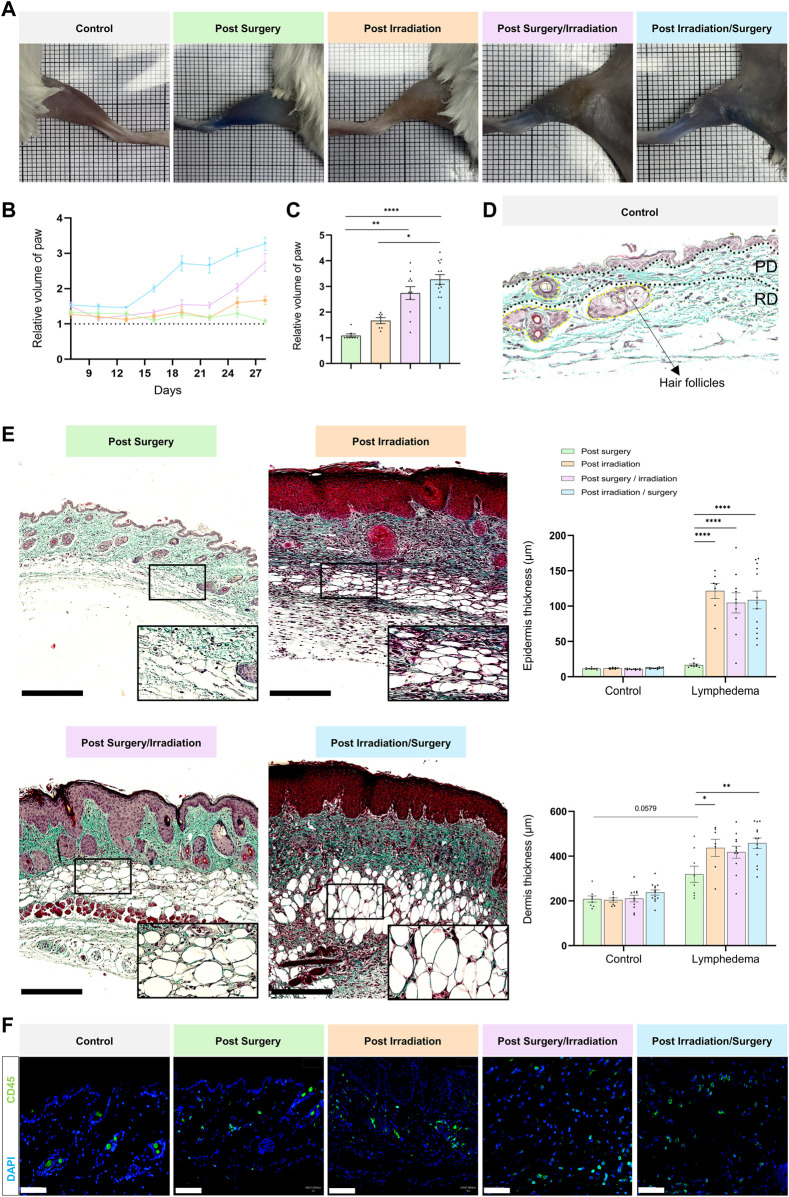
Macroscopic and histological characterization of lymphedema. Experimental groups are defined in Figure 1. **(A)** Representative images of control (right paw) and LD (left paw) limbs at day 28. **(B)** Quantification of the relative volume of LD to control limbs for the four groups over time. **(C)** Bar graph showing relative paw volume at D28 (statistical test: one-way ANOVA Kruskal–Wallis test, *p* < 0.05: *, *p* < 0.01: **, *p* < 0.001: ***, *p* < 0.0001: ****). **(D)** Representative image of a paraffin-embedded control skin section stained with Masson’s trichrome. The epidermis, papillary dermis (PD), reticular dermis (RD) and hair follicles are delineated. Scale bar = 100 µm. **(E)** Representative images of paraffin-embedded LD skin sections stained with Masson trichrome for each group. Scale bar: 250 µm. The black rectangle represents the magnification (×30) on adipocytes. Graphs on the right correspond to epidermal (top) and dermal (bottom) thicknesses in control and LD skin from each group (Statistical test: two-way ANOVA with multiple comparison Turkey’s test, *p* < 0.05: *, *p* < 0.01: **, *p* < 0.001: ***, *p* < 0.0001: ****). **(F)** Presence of CD45 positive inflammatory cells within the LD tissue of each group (scale bar: 50 µm).

Procedures and timelines used to induce LD are depicted in Figure 1. Mice were separated into four experimental groups and subjected either to: 1) surgery alone (group 1: “post-Surgery” LD) (*n* = 8); 2) irradiation alone (group 2: “post-Irradiation” LD) (*n* = 7); 3) surgery followed by irradiation (group 3: “post-Surgery/Irradiation” LD) (*n* = 11); or 4) irradiation followed by surgery (group 4: “post-Irradiation/Surgery” LD) (*n* = 13). Days 0 and seven refer to the day of the first and second steps of the LD induction procedure (either surgery or irradiation as indicated in Figure 1), respectively. For all animals, the left hind limb was subjected to LD induction, while the right hind limb was not treated and used as an internal control (Figure 1). Limb swelling was macroscopically discernible (Figure 2A) 16 and 25 days after the initiation of LD induction in “post-Irradiation/Surgery” LD (group 4) and “post-Surgery/Irradiation” LD (Group 3), respectively (Figure 2B). Almost all mice (at least >95%) of these experimental groups exhibited limb swelling. At the end of the assay, limb volumes reached to 2,5 to 3-fold the size of the corresponding control limb (Figure 2C). These data indicate that swelling occurred similarly in animals subjected to a combination of irradiation and surgery, independently of the sequence (groups 3 and 4). It is, however, noteworthy that it appeared faster in group 4 when irradiation preceded surgery. The single irradiation or surgery did not affect or slightly increased the limb volume without reaching statistical significance (Figures 2B,C).

The macroscopic observations were confirmed by histological examination of tissue sections (Figures 2D,E). The epidermis and the dermis were thicker in LD skins of groups 3 and 4, as compared to the contralateral normal skins. Interestingly, epidermal and dermal thickening occurred also in LD skins of irradiated mice (group 2). In sharp contrast, the single surgical procedure (group 1) affected very slightly the thickness of epidermis and dermis. These data support that two important LD hallmarks (epidermis hyperplasia and dermis thickening) were displayed in LD skins induced by irradiation (combined or not with surgery). Of note, a drastic accumulation of adipocytes and inflammatory cells was also noticed in LD areas induced by irradiation combined to surgery (and *vice versa*) (Figures 2E,F). These cells were also detected, but to a lesser extend in LD induced by single irradiation or surgery. Altogether, these data provide evidence for a severe LD induction by a combination of irradiation and surgical procedure, as assessed by five LD features (limb swelling, epidermis hyperplasia, dermis thickening, adipocyte accumulation and inflammatory cell infiltration).

**FIGURE 3:**
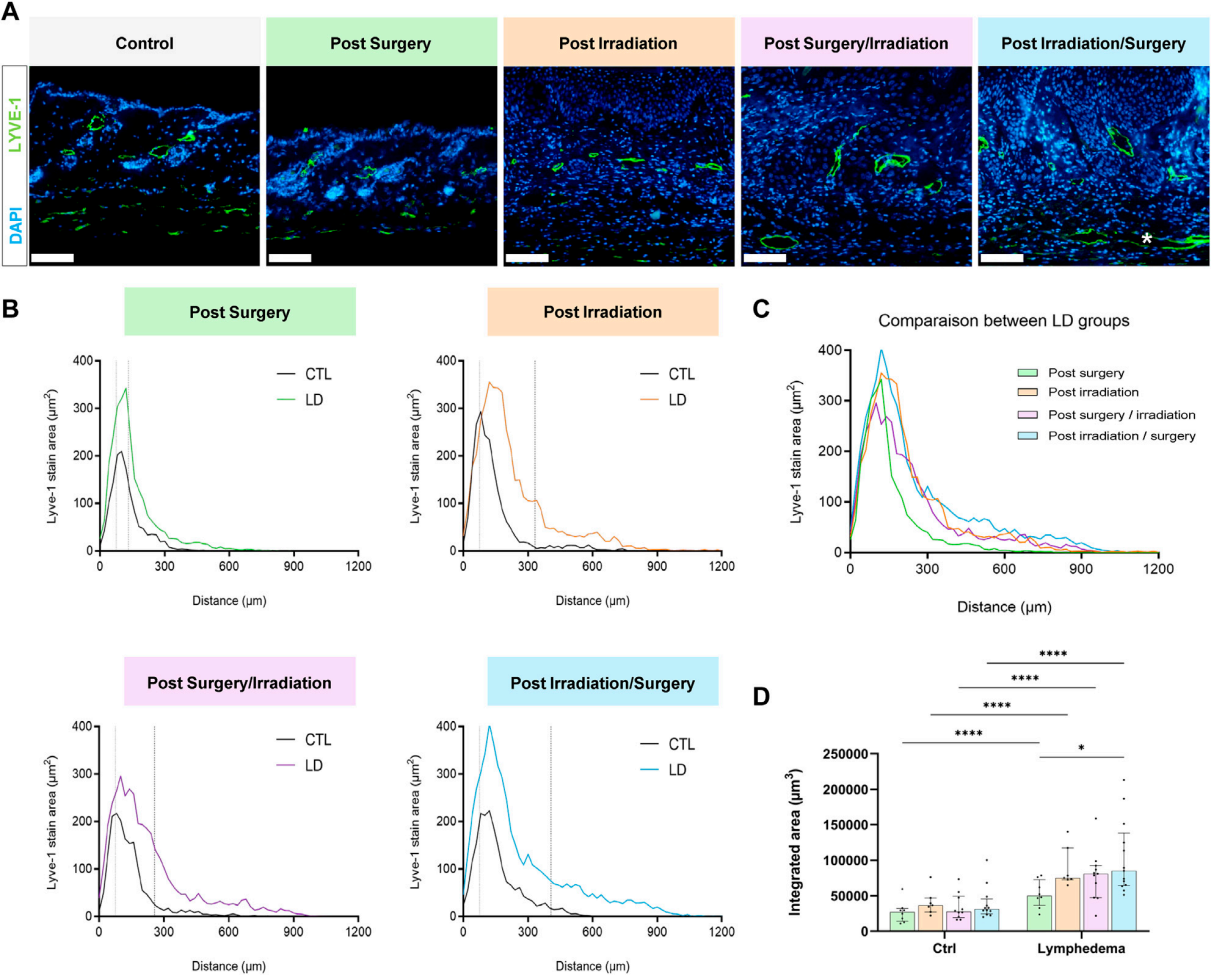
Impact of the procedures on lymphatic vasculature. **(A)** Representative images of Lyve-1 positive vessels in control and LD skins. * White asterisk highlights large lymphatic vessels in the reticular dermis (Scale bar: 100 µm). **(B)** Spatial distribution of Lyve-1 positive lymphatic vessels within the dermis, 0 on the *x*-axis corresponds to the junction between dermis and epidermis. The dotted line delineates the junction between papillary (PD) and reticular (RD) dermis. **(C)** Comparison of lymphatic vessel distribution curves between the different experimental groups. **(D)** Quantification of total labeling area in the dermis determined by measuring the area under the distributions curve (“Integrated Area”) (Statistical test: two-way ANOVA with multiple comparison Turkey’s test, *p* < 0.05: *, *p* < 0.01: **, *p* < 0.001: ***, *p* < 0.0001: ****).

**FIGURE 4:**
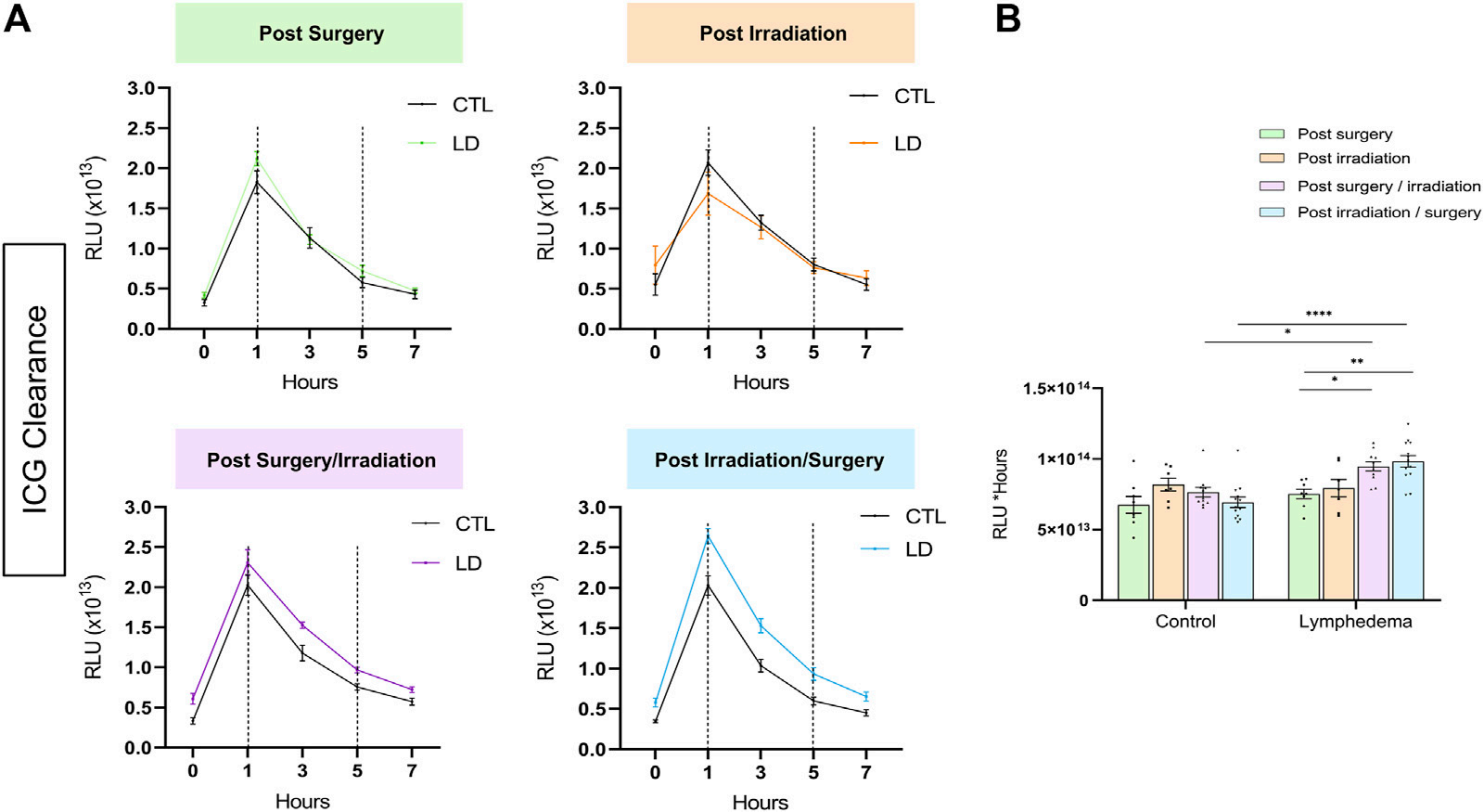
Tracking of ICG fluorescence resorption over time in lymphedema. **(A)** ICG is accumulated in the LD of the post-Surgery/Irradiation and Irradiation/Surgery groups. **(B)** Comparison of areas under the curve between 1 h (onset of clearance) and 5 h between the different groups. (Statistical test: two-way ANOVA with multiple comparison Turkey’s test, *p* < 0.05: *, *p* < 0.01: **, *p* < 0.001: ***, *p* < 0.0001: ****).

In a normal skin, lymphatic capillaries detected by Lyve-1 immunostaining are mainly localized around hair follicles in the papillary dermis. A deeper skin lymphatic plexus composed of collecting vessels that are negative for Lyve-1 is not detected through the immunostaining used here. Accordingly, Lyve-1+ lymphatic vessels were primarily confined to the papillary dermis of control skins resected from the right limbs. In LD skins, Lyve-1 positive lymphatic capillaries were detected in the papillary dermis, as well as deeper in the reticular dermis (Figure 3). Of note, lymphatic vessels were dilated in the reticular dermis of groups 3 and 4 subjected to both interventions (Figure 3A). A digital image analysis approach was applied to characterize the lymphatic vasculature on whole scanned sections, as previously described (Balsat et al., 2017). A computer-assisted method was then used to determine the spatial distribution of lymphatic vessels (Balsat et al., 2014; Balsat et al., 2017) (Figures 3B–D). In control skins, 90% of lymphatic vessels (P_90_: 90th percentile) were detected through a distance ranging from 225 to 300 um from the epithelial basal layer. A similar distribution of lymphatic vessels was seen in “post-Surgery” LD (group 1) skins (P_90_ at a distance of 292 um). In the other LD groups (groups 2-4), lymphatic vessels infiltrated deeper into the dermis with P_90_ found at a twice longer distance from the epithelium (P_90_ at a distance ranging from 600 to 800 um). The determination of the surface occupied by lymphatic capillaries (“integrated area”) revealed an enhancement of lymphatic vessel formation in all LD groups, with more pronounced effects in group 4 (3-fold increase in group 4 as compared to their corresponding control, instead 2-fold increase in groups 1, 2 and 3) (Figure 3D). Altogether, these data point out a modification of lymphatic vasculature in the dermis that is induced by the combination of irradiation and surgery. To correlate the observed lymphatic remodeling with fluid drainage capacity, we analyzed the clearance of indocyanine green (ICG) intradermally injected, at day 27, in mouse hind foot paw (Figure 4). A slow decrease of the fluorescent signal observed from 1 to 7 h post ICG injection reflected a progressive clearance of the tracer (Figure 4A). The evolution of the fluorescent signal intensity overtime was determined by calculating the area under the curve (“integrated area” from 1 to 5 h) (Figure 4B). In groups 1 and 2 subjected to single procedure (either irradiation or surgery), ICG clearance was similar in left and right limbs. In contrast, the ICG accumulation was higher in LD (left) limbs than in control (right) limbs of mice subjected to combined treatments. This indicates that ICG clearance was less efficient in LD limbs than in normal limbs suggesting that lymphatic functionality was impaired in groups 3 and 4.

**FIGURE 5:**
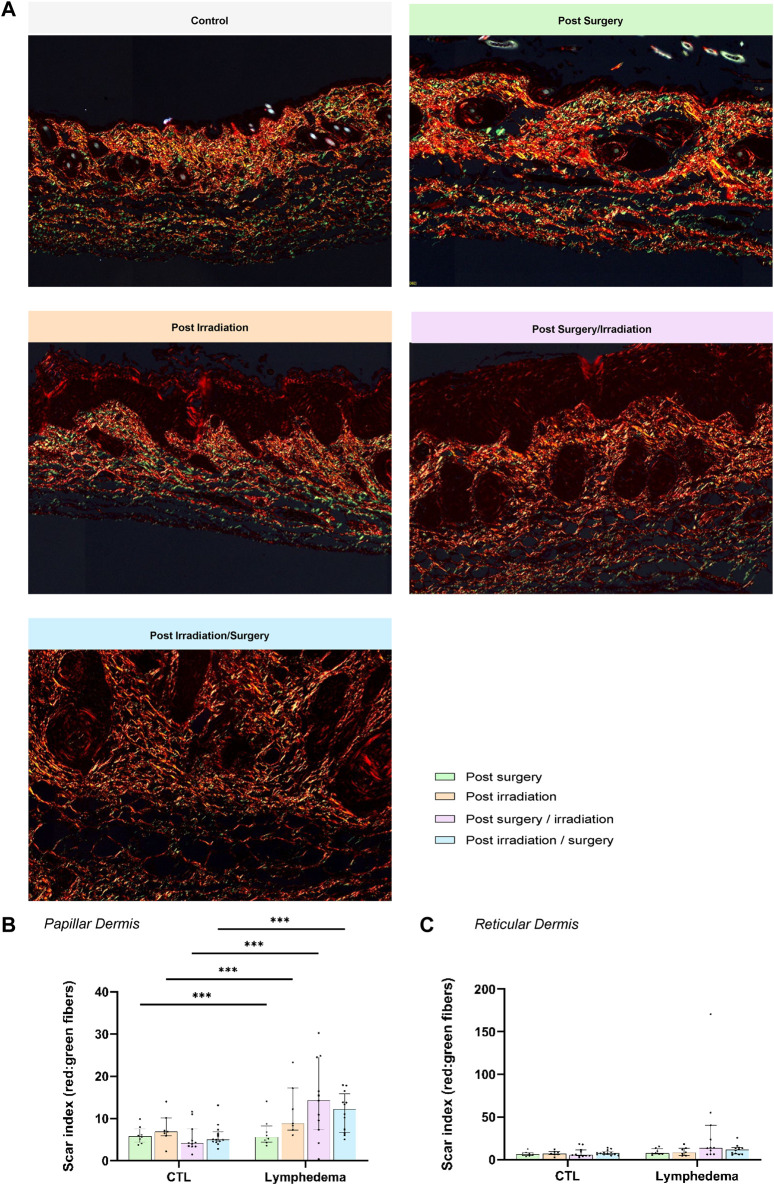
Impact of the procedures on collagen deposition. **(A)** Representative images of red Sirius visualized through polarized light in control and LD skins (Scale bar: 200 µm). **(B)** and **(C)** Scar Index (red/green staining’s) calculated through computerized method in PD **(B)** and RD **(C)**. The dotted line delineates the junction between PD and RD.

**FIGURE 6:**
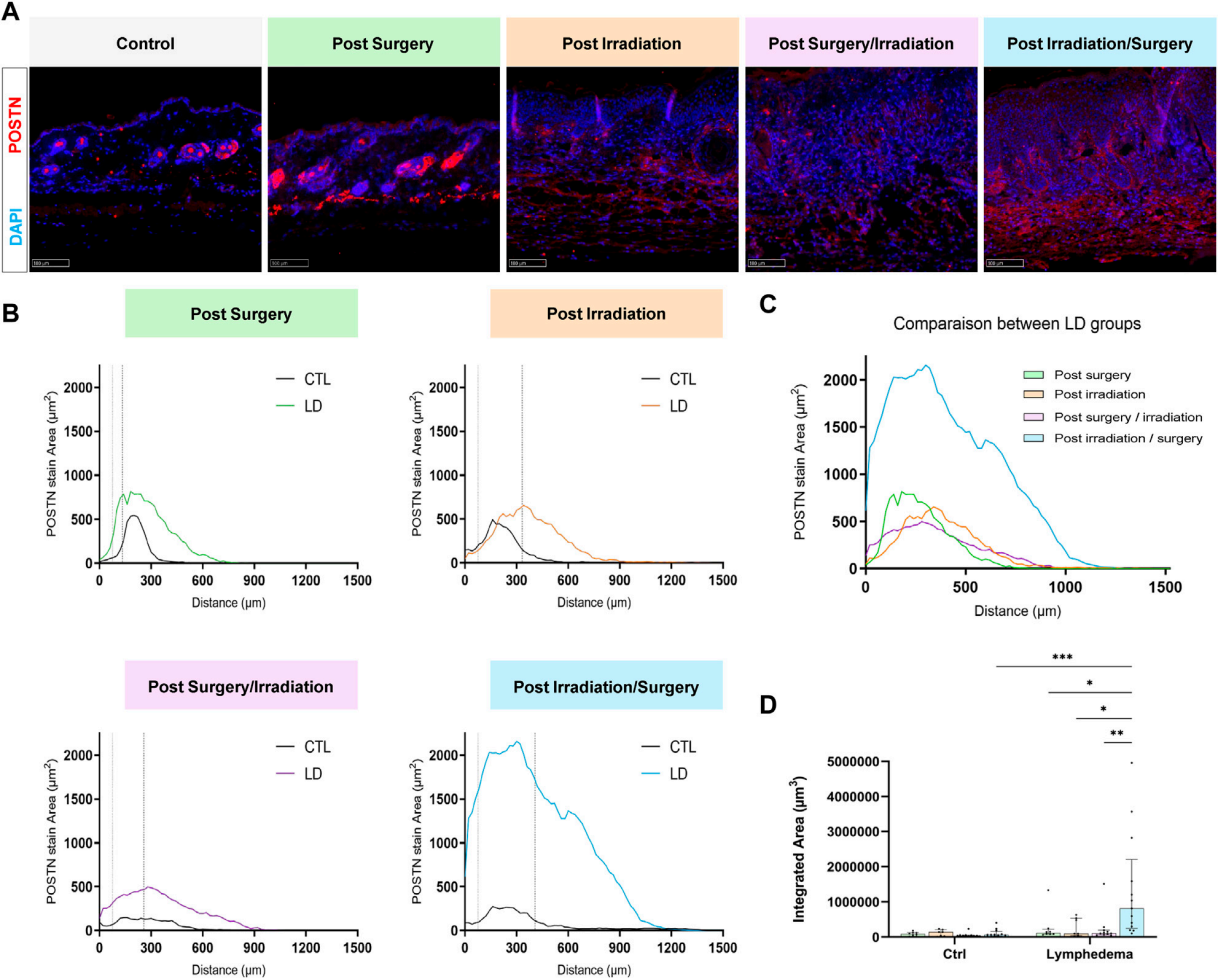
Impact of the procedures on POSTN deposition. **(A)** Representative images of POSTN area staining in control and LD skins (Scale bar: 100 µm). **(B)** Distribution curves of POSTN (*X* axis correspond to the distance from the epidermis/dermis junction). The dotted line delineates the junction between papillary and reticular dermis (PD and RD, respectively). **(C)** Comparison of the distribution curves of POSTN staining in the different groups. **(D)** Quantification of total labeling area in the dermis determined by measuring the area under the distributions curve (“Integrated Area”) (Statistical test: two-way ANOVA with multiple comparison Turkey’s test, *p* < 0.05: *, *p* < 0.01: **, *p* < 0.001: ***, *p* < 0.0001: ****).

**FIGURE 7:**
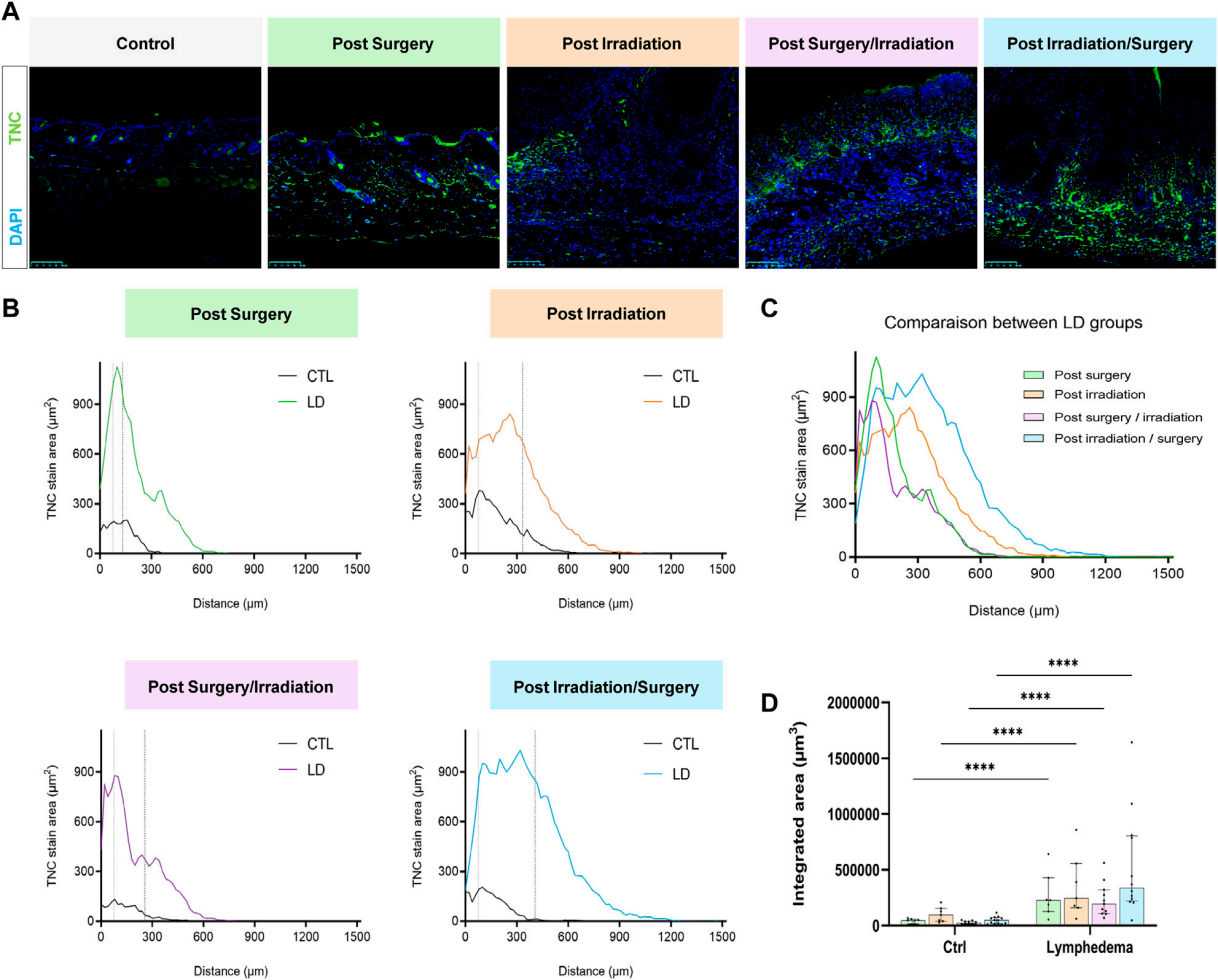
Impact of the procedures on TNC deposition. **(A)** Representative images of TNC area staining in control and LD skins (Scale bar: 100 µm). **(B)** Spatial distribution of TNC within the dermis (0 on the *x*-axis is the junction between dermis and epidermis). The dotted line delineates the junction between papillary (PD) and reticular (RD) dermis. **(C)** Comparison of the distribution of TNC staining area between experimental groups. **(D)** Quantification of total labeling area in the dermis determined by measuring the area under the distributions curve (“Integrated Area”) (Statistical test: two-way ANOVA with multiple comparison Turkey’s test, *p* < 0.05: *, *p* < 0.01: **, *p* < 0.001: ***, *p* < 0.0001: ****).

We next analyzed red Sirius staining under polarized light to estimate the organizational pattern of collagen by detecting thick (red-orange staining) and thin (weekly birefringent green staining) fibers corresponding mainly to type I and type III collagen, respectively. Increased collagen deposition was found in all LD groups (Figure 5A). All intervention procedures led to increased scar index (thick/thin fiber ratio) in papillary dermis (Figure 5B), but not in reticular dermis (Figure 5C). Importantly, a 1.5-fold enhancement of scar index was found in group 2 subjected to Irradiation alone and two to 3-fold enhancement of scar index in groups 3-4 (subjected to the combination of the treatments), reflecting a scarring fibrosis. Since matricellular proteins are known as nonstructural proteins that can interact with collagen and modulate cell functions, we next investigated two of them, periostin (POSTN) and tenascin-C (TNC) (Figures 6, 7). POSTN and TNC are both reported to be weakly expressed in quiescent adult tissues but strongly expressed under fibrotic conditions. As expected in normal skin, POSTN was produced in papillary dermis and around hair follicles (Figure 6A). The determination of POSTN spatial distribution revealed a faint deposition under a distance of 300–400 um (P_90_) from the epidermis (Figures 6B,C). In all experimental groups, POSTN staining extended deeper into the dermis of limb subjected to LD induction (P_90_ ranging from 600 to 1,000 um). The surface occupied by POSTN staining was also enhanced in LD skins of groups 1, 2 and 3 (2 to 4-fold enhancement as compared to their controls). Importantly, the modification of POSTN staining was much more pronounced in group 4, in which the global surface of positive area was 13-fold increased (Figure 6D). Interestingly, we found a positive correlation between POSTN and Lyve-1 staining areas (Spearman coefficient *r* ^2^ = 0,71, *p* < 0.008). Although a faint staining was detected in control skins, TNC deposition was markedly induced by the different procedures. The distribution and surface of TNC signals were almost similar in groups 1, 2 and 3 (Figures 7B–D). Again, “post-Irradiation/Surgery” LD were characterized by a 11-fold enhancement of TNC staining surface.

## Discussion

We here investigated the respective implication of surgery and irradiation on LD development and features by applying these treatments to the hind limb of mice, alone or in combination with different sequences of intervention. We provide evidence that irradiation followed by surgery led to the most rapid onset of a severe LD that sustains during at least 3 weeks. Macroscopic, histological and functional analyses demonstrated that the induced LD featured all hallmarks of secondary LD with swelling, epidermal hyperplasia, dermal thickening, lymphatic vasculature remodeling with vessel dilatation and reduced drainage function, and fibrosis associated to adipocyte accumulation and inflammation. In contrast, mild LD lacking some disease characteristics were generated by single interventions (either irradiation or surgery). We also provide the first experimental evidence of POSTN and TNC deposition in the dermis of LD skins. Through a computerized method applied to digital images, we established a map of the lymphatic vasculature and of structural (collagen) and matricellular (POSTN and TNC) proteins in the papillary and reticular dermis of LD skins.

Secondary LD in cancer patients can be initiated by lymph node excision performed for staging purposes and/or to prevent metastatic dissemination. It is thus believed that LD arises due to the lymphatic damage serving as the initiator of a cascade of events. In this context, the most widely used murine tail model for investigating LD is based on lymphatic stasis induced by surgical removal of a superficial layer of mice tail and lymph vessel ligation (Rutkowski et al., 2006). This model has proven its suitability to study the pathophysiology of LD (Gardenier et al., 2017; Choi et al., 2019; Weiler et al., 2019; Hassanein et al., 2021). To better mirror the clinical situation, we here applied irradiation and surgery to mice limb. Surgery alone was not sufficient to induce a severe LD in hind limb, even though we applied a radical surgery (combining popliteal and inguinal lymph node ablation with lymphatic vessel ligation) in order to create a steady deterioration of the lymphatic system. Despite the lack of discernable limb swelling, the single surgical intervention led to a moderate tissue remodeling displaying part of the LD features that are detectable through histological examination (epidermis hyperplasia, lymphatic expansion and fibrosis). Lymphatic damage is thus an initiating, but insufficient event to generate LD associated to swelling. This is in line with the fact that LD does not develop uniformly in all patients who undergo lymphadenectomy, but rather only in a subset of them. Radiotherapy together with BMI, age, tumor stages, lymph node dissection and the number of lymph nodes removed are the main risk factors for lymphedema after cancer treatment (Tsai et al., 2009; DiSipio et al., 2013; Warren et al., 2014; Hu et al., 2022). Radiotherapy is well known to cause tissue fibrosis, as a result of transforming growth factor TGF-β1 production (Martin et al., 2000; Baik et al., 2022). It also affects lymphatic function by inducing lymphatic endothelial cell apoptosis and decreasing the number of cutaneous lymphatic vessels (Avraham et al., 2009). Accordingly, in our model, irradiation combined to surgery was essential to cause a rapid onset of a severe and persisting hind limb LD. Surgery contributes to minimal LD development (group 1). Irradiation alone (group 2) did not induce drastic swelling, but affected the other LD features analyzed. Indeed, radiation-induced skin fibrosis is a known event observed in clinical practice. The optimal sequence of intervention was irradiation prior to surgery (group 4). The inverse sequence (group 3) led to a slower onset of LD, nevertheless inducing limb size changes at the end of the assay, associated to reduced fibrosis and lymphatic expansion. The impact of intervention sequence could be partly explained by a shorter period between irradiation and LD skin resection and histological examination, in group 3 (2 weeks instead of 3 weeks in group 4). In group 4, the irradiation which precedes surgery can induce an early release of cytokines that promote inflammation, fibrosis and vascular remodeling. These processes are then likely exacerbated by the subsequent surgical intervention, accounting for the higher swelling and LD development noted in group 4. Together, these data further support the importance of a cascade of pathologic modifications to experimentally induce different features of the disease.

LD generated in groups 3 and 4 subjected to combined interventions was associated *in vivo* with increased accumulation and slower clearance of a tracer (ICG), confirming the edema formation. Once lymphatic vascular dysfunction becomes established through surgical intervention, the subsequent interstitial edema can induce a lymphatic vascular remodeling. This enhanced lymphatic formation is expected to contribute to LD resolution. One interesting finding of our study is the increased lymphangiogenesis occurring deeply into the reticular dermis. In the normal skin, lymphatic vasculature is composed of two plexuses (Skobe and Detmar, 2000; Lund et al., 2016). The superficial one is located in the papillary dermis where lymphatic capillaries form an intimate network with hair follicle stem cells and are a crucial element of the stem cell niche (Gur-Cohen et al., 2019). The deeper plexus extending near the subpapillary arterial vasculature (Skobe and Detmar, 2000) are mainly composed of Lyve-1 negative collecting vessels containing valves that prevent retrograde fluid flow. Though a computerized digital image quantification, we provide a map of lymphatic capillaries revealing their extension into the reticular dermis. Importantly, lymphatic capillaries infiltrated over a distance twice higher in groups subjected to irradiation. These data highlight the multiple and complex effects of irradiation that induce tissue damage and a reactive response potentially resulting in lymphatic expansion.

Tissue fibrosis is a key step in LD development and its degree correlates with the severity of disease (Hu et al., 2022). It is a key hallmark of a late disease state, as abnormal collagen deposition takes time to accumulate. Through Sirius red staining, we provided evidence for scarring fibrosis in papillary dermis of LD skins, particularly in animals of groups 2 and 4. An original finding of the present study is the detection of two matricellular proteins (TNC and POSTN) in LD skins. Their role in LD is not documented yet, although the expression of their genes have been reported through transcriptomic analyses of human LD samples (Planck et al., 2011). To the best of our knowledge, we here provide the first experimental evidence for an excessive production of the two proteins in LD. While POSTN and TNC were not or faintly detected in normal skins, a drastic enhancement of their deposition was found in LD. The most important effect was again observed in “post-Irradiation/Surgery” LD (group 4). Such an excessive deposition under a pathological condition is in line with previous reports (Gonzalez-Gonzalez and Periostin, 2018; Nikoloudaki et al., 2020; Gillot et al., 2022). Mice-deficient for POSTN or TNC developed normally and exhibited no major phenotypes in adulthood suggesting a limited implication under physiological conditions (Saga et al., 1992; Forsberg et al., 1996; Murphy-Ullrich and Sage, 2014). In sharp contrast when injured, those mice exhibited significant defects in healing, which highlights an important role for matricellular proteins in wound-healing and fibrotic processes (Kasprzycka et al., 2015). Whereas type I collagen provides a structural support to tissues, matricellular proteins are most often considered as non-structural proteins that regulate several cell functions (adhesion, spreading, migration, proliferation, and differentiation), at least by interacting with cell surface receptors (e.g. integrins). POSTN is a pro-fibrotic protein expressed in skin fibrotic scars, upregulated during cutaneous wound repair where it promotes myofibroblast differentiation (Nikoloudaki et al., 2020). In a translational and pre-clinical study, we recently demonstrated the key implication of POSTN in promoting the lymphangiogenic response associated to pre-metastatic niche elaboration in human and murine lymph nodes (Gillot et al., 2021; Gillot et al., 2022). We provided *in vitro* evidence that POSTN promoted lymphatic endothelial cell adhesion, spreading, migration and proliferation. Furthermore, the *in vivo* injection of recombinant POSTN together with VEGF-C stimulated lymphangiogenesis in lymph node revealing that POSTN can boost the lymphangiogenic response initiated by VEGF-C (Gillot et al., 2021; Gillot et al., 2022). In line with these findings, we noted a positive correlation between lymphatic vessel and POSTN staining’s where the LD was more pronounced (group 4, “post-Surgery/Irradiation” LD). POSTN can directly bind collagen I *via* its EMI domain and contributes to collagen fibrillogenesis, collagen cross-linking, and the formation of a complex matrix meshwork *via* interactions with other matrix components (Norris et al., 2007; Kii and Ito, 2017). Interestingly POSTN can also interact with TNC through its FAS1 domains. TNC is associated with fibrotic pathologies and its role in fibrosis is well documented Bhattacharyya (Bhattacharyya et al., 2016). This protein is particularly important and acts both on tissue remodeling and on chronic inflammation allowing the maintenance of fibrosis. TNC can upregulate collagen production, at least in part by inducing TGF-β1 and activating SMAD2/3 signaling pathway through the TGF-β receptors (Choi et al., 2020). Altogether our data highlight the complex interplay between the different matrix components. They also demonstrate that beyond quantifying collagen deposition, qualitative analyses of matrix composition is worth performing to accurately characterize LD-associated fibrosis.

Currently, treatments of LD involve palliative (compression garments and physical therapy) and surgical approaches (liposuction, lymphovenous bypass and vascularized lymph node transfer). Efficient pharmacologic treatments are mandatory to avoid the need for surgery in chronic and severe LD associated to fibrosis. Targeting matrix remodeling could provide novel opportunities to prevent or manage LD (Kataru et al., 2019). The murine LD model optimized in this study has the advantage to be close to the clinical context by combining surgery and irradiation It also provides the possibility to study the relationship between lymphatic vasculature and its surrounding fibrotic tissue. The 3–4 weeks temporal window offered by “post-Irradiation/Surgical” LD is suitable for mechanistic investigations and drug testing. Experimental murine LD, including the present one, typically resolves within about 1 month whereas LD in humans worsens over time. This could be seen as an important model limitation. On the other side, one can view it as an opportunity to study the resolution process of LD in mice.

## Data Availability

The raw data supporting the conclusion of this article will be made available by the authors, without undue reservation.
